# Supplementary material for: Inheritance of Early and Late Ascochyta Blight Resistance in Wide Crosses of Chickpea
Source: Genes (Basel). 2023 Jan 26;14(2):316. doi: 10.3390/genes14020316 (PMC9957483; doi:10.3390/genes14020316)
Supplement: Supplementary file 1 [file genes-14-00316-s001.zip › Supporting Table S1.pdf]

Table S1. Comparison of AUDPC of different sample types between the experiments for the Gokce x Oyali and Gokce x Karab families.

Comparison	U statistic	p value	adj. pvalue
Gocke	12	3.09E-01	3.87E-01
Wild parent	13	1.91E-01	3.19E-01
SusCheck	16	2.86E-02	7.14E-02
ResCheck	9	8.86E-01	8.86E-01
Family	16833	3.84E-12	*1.92E-11*

Notes. Comparisons are the Gokce parents, Wild parent = Oyali and Karab, SusCheck = susceptible check lines, ResCheck = resistant check lines, Family = F2 hybrids for each family. Summary U statistics and p values for Mann-Whitney-Wilcoxon tests are shown. P value adjustment by Benjamini-Hochberg method was applied. P values in italics show significant differences after adjustment.
