# Supplementary material for: Inheritance of Early and Late Ascochyta Blight Resistance in Wide Crosses of Chickpea
Source: Genes (Basel). 2023 Jan 26;14(2):316. doi: 10.3390/genes14020316 (PMC9957483; doi:10.3390/genes14020316)
Supplement: Supplementary file 1 [file genes-14-00316-s001.zip › Supporting Table S3.pdf]

Table S3. All chickpea genes found within a 200k bp region around significant Ascochyta resistance quantitative trait loci in the chickpea Gokce x Oyali and Gokce x Karab families.

QTL	Name	Location	UniProt Homology	InterPro Annotation
GK 21 days DS	Ca_10198	Ca2: 33290766 .. 33292184	I3SF84_LOTJA Uncharacterized protein OS=Lotus japonicus PE=4 SV…	IPR001163: LSM_dom_euk/arc
GK 21 days DS	Ca_10197	Ca2: 33297823 .. 33302123	GOT1_ARATH Vesicle transport protein GOT1 OS=Arabidopsis thal...	IPR007305: Vesicle_transpt_Got1/SFT2
GK 21 days DS	Ca_10196	Ca2: 33308505 .. 33317020	RGAP7_ARATH Rho GTPase-activating protein 7 OS=Arabidopsis tha…	IPR000198: RhoGAP_dom

GK 21 days DS	Ca_10195	Ca2: 33326287 .. 33326592	G7K326_MEDTR Putative uncharacterized protein OS=Medicago trunc…	
GK 21 days DS	Ca_10194	Ca2: 33327566 .. 33330351	VA711_ARATH Vesicle-associated membrane protein 711 OS=Arabido...	IPR001388: Synaptobrevin
GK 21 days DS	Ca_10193	Ca2: 33333506 .. 33337684	CLT3_ARATH Protein CLT3, chloroplastic OS=Arabidopsis thalian…	
GK 21 days DS	Ca_10192	Ca2: 33339116 .. 33342106	STR12_ARATH Rhodanese-like/PpiC domain-containing protein 12, ...	IPR000297: PPIase_PpiC
GK 21 days DS	Ca_10191	Ca2: 33344213 .. 33347181	CDPKH_ARATH Calcium-dependent protein kinase 17 OS=Arabidopsis...	IPR000719: Prot_kinase_dom

GK 21 days DS	Ca_10190	Ca2: 33349755 .. 33350129	SF3B6_ARATH Splicing factor 3B subunit 6-like protein OS=Arabi...	IPR000504: RRM_dom
GK 21 days DS	Ca_10189	Ca2: 33352996 .. 33354461	K7M9T1_SOYBN Uncharacterized protein OS=Glycine max PE=4 SV=1	IPR008700: TypeIII_avirulence_cleave
GK 21 days DS	Ca_10188	Ca2: 33355180 .. 33360490	DPYS_ARATH Dihydropyrimidinase OS=Arabidopsis thaliana OX=370...	IPR011059: Metal-dep_hydrolase_composite
GK 21 days DS	Ca_10187	Ca2: 33367242 .. 33373022	G7KCV9_MEDTR LAS1-like protein OS=Medicago truncatula GN=MTR_5g..	IPR007174: Las1
GK 21 days DS	Ca_10186	Ca2: 33398850 .. 33402874	R13L1_ARATH Putative disease resistance RPP13-like protein 1 O…	IPR000767: Disease resistance protein

GK 21 days DS	Ca_10185	Ca2: 33428729 .. 33430537	G7I3Q0_MEDTR Putative uncharacterized protein OS=Medicago trunc...	
GK 7 days DS	Ca_07392	Ca3: 30712266 .. 30716068	IDM1_ARATH Increased DNA methylation 1 OS=Arabidopsis thalian…	
GK 7 days DS	Ca_07393	Ca3: 30719535 .. 30720260	OFP16_ARATH Transcription repressor OFP16 OS=Arabidopsis thali…	IPR006458: Ovate_C
GK 7 days DS	Ca_07394	Ca3: 30731787 .. 30732398	I1JF92_SOYBN Uncharacterized protein OS=Glycine max PE=4 SV=1	
GK 7 days DS	Ca_07395	Ca3: 30744451 .. 30748192	K7MYL5_SOYBN Uncharacterized protein OS=Glycine max PE=4 SV=1	IPR001680: WD40_repeat

GK 7 days DS	Ca_07396	Ca3: 30750663 .. 30751715	SKI30_ARATH F-box/kelch-repeat protein SKIP30 OS=Arabidopsis t...	IPR006652: Kelch_1
GK 7 days DS	Ca_07397	Ca3: 30756384 .. 30757023	NLTL2_ARATH Non-specific lipid-transfer protein-like protein A...	IPR000528: Plant_nsLTP
GK 7 days DS	Ca_07398	Ca3: 30764244 .. 30767623	IP5P7_ARATH Type IV inositol polyphosphate 5-phosphatase 7 OS=…	IPR005135: Endo/exonuclease/phosphatase
GK 7 days DS	Ca_07399	Ca3: 30772506 .. 30776508	HMT2_ARATH Homocysteine S-methyltransferase 2 OS=Arabidopsis	IPR003726: HCY_dom
GK 7 days DS	Ca_07400	Ca3: 30788023 .. 30794359	NET1D_ARATH Protein NETWORKED 1D OS=Arabidopsis thaliana OX=37…	IPR011684: NAB

GK 7 days DS	Ca_07401	Ca3: 30798652 .. 30805732	SUT31_ARATH Sulfate transporter 3.1 OS=Arabidopsis thaliana GN…	IPR001902: SLC26A/SulP_fam
GK 7 days DS	Ca_12181	Ca3: 30835030 .. 30839197	VAB_ARATH VAN3-binding protein OS=Arabidopsis thaliana OX=37...	IPR008546: DUF828
GK 7 days DS	Ca_12182	Ca3: 30839471 .. 30840902	LFG4_ARATH Protein LIFEGUARD 4 OS=Arabidopsis thaliana OX=370...	IPR006214: Bax_inhibitor_1-related
GK 7 days DS	Ca_12183	Ca3: 30859526 .. 30861271	HFA6B_ARATH Heat stress transcription factor A-6b OS=Arabidops…	IPR000232: HSF_DNA-bd
GK 7 days DS	Ca_12184	Ca3: 30868014 .. 30869498	P24B3_ARATH Transmembrane emp24 domain-containing protein p24b…	IPR009038: GOLD_dom

GK 7 days DS	Ca_12185	Ca3: 30879257 .. 30882427	ACA12_ARATH Calcium-transporting ATPase 12, plasma membrane-ty…	IPR000695: ATPase, P-type, H+ transporting proton pump
GK 7 days DS	Ca_12186	Ca3: 30896488 .. 30900928	CYP63_ARATH Peptidyl-prolyl cis-trans isomerase CYP63 OS=Arabi..	IPR002130: Cyclophilin-type_PPIase_dom
GK 7 days DS	Ca_12187	Ca3: 30905629 .. 30909158	K7K8C2_SOYBN Uncharacterized protein OS=Glycine max PE=4 SV=1	IPR025486: DUF4378
GK 14 days DS	Ca_05907	Ca6: 3940697 .. 3941854	C3H49_ARATH Zinc finger CCCH domain-containing protein 49 OS=A...	IPR000571: Znf_CCCH
GK 14 days DS	Ca_05906	Ca6: 3951194 .. 3954510	SYNC1_ARATH Asparagine--tRNA ligase, cytoplasmic 1 OS=Arabidop…	IPR000738: WHEP-TRS_dom

GK 14 days DS	Ca_05905	Ca6: 3959326 .. 3962494	VP322_ARATH Vacuolar protein sorting-associated protein 32 hom...	IPR005024: Snf7_fam
GK 14 days DS	Ca_05904	Ca6: 3965401 .. 3968398	VP322_ARATH Vacuolar protein sorting-associated protein 32 hom…	IPR005024: Snf7_fam
GK 14 days DS	Ca_05903	Ca6: 3976076 .. 3977244	RL311_ARATH 60S ribosomal protein L31-1 OS=Arabidopsis thalian...	IPR000054: Ribosomal_L31e
GK 14 days DS	Ca_05902	Ca6: 3980226 .. 3984276	PQQL_ARATH Zinc protease PQQL-like OS=Arabidopsis thaliana OX...	
GK 14 days DS	Ca_05901	Ca6: 3989387 .. 3992551	PQQL_ARATH Zinc protease PQQL-like OS=Arabidopsis thaliana OX…	IPR001431: Pept_M16_Zn_BS
GK 14 days DS	Ca_05900	Ca6: 3993752 .. 3995748	FBL77_ARATH F-box/LRR-repeat protein At4g29420 OS=Arabidopsis …	
GK 14 days DS	Ca_05899	Ca6: 3997817 .. 3998769	R15A5_ARATH 40S ribosomal protein S15a-5 OS=Arabidopsis thalia...	IPR000630: Ribosomal_S8

GK 14 days DS	Ca_05898	Ca6: 3999495 .. 4001208	CAMT3_ARATH (Probable caffeoyl-CoA O-methyltransferase At4g26220	IPR002935: SAM_O-MeTrfase
GK 14 days DS	Ca_05897	Ca6: 4002176 .. 4005530	A9PAP2_POPTR Predicted protein OS=Populus trichocarpa GN=POPTRD...	IPR006808: ATP_synth_F0_gsu_mt
GK 14 days DS	Ca_05896	Ca6: 4007544 .. 4008870	I3S351_LOTJA Uncharacterized protein OS=Lotus japonicus PE=2 SV...	IPR006808: ATP_synth_F0_gsu_mt
GK 14 days DS	Ca_05895	Ca6: 4031335 .. 4032971	1A17_ARATH 1-aminocyclopropane-1-carboxylate synthase 7 OS=Ar...	IPR001176: 1-aminocyclopropane-1-carboxylate synthase
GK 14 days DS	Ca_05894	Ca6: 4042490 .. 4048684	I1K635_SOYBN Uncharacterized protein OS=Glycine max PE=3 SV=1	IPR000994: Pept_M24
GK 14 days DS	Ca_05893	Ca6: 4051006 .. 4053342	NDL1_ARATH Protein NDL1 OS=Arabidopsis thaliana OX=3702 GN=ND...	IPR004142: NDRG
GK 14 days DS	Ca_05892	Ca6: 4059713 .. 4061288	PPSP3_ARATH Thiamine phosphate phosphatase-like protein OS=Ara...	IPR006383: HAD-SF_hydro_IB_PSP-like

GK 14 days DS	Ca_05891	Ca6: 4066273 .. 4070335	PERK9_ARATH Proline-rich receptor-like protein kinase PERK9 OS...	IPR000719: Prot_kinase_dom
GK 14 days DS	Ca_05890	Ca6: 4075678 .. 4076547	CDA1_ARATH Cytidine deaminase 1 OS=Arabidopsis thaliana OX=37...	IPR002125: CMP_dCMP_dom
GK 14 days DS	Ca_05889	Ca6: 4083942 .. 4085225	KUA1_ARATH Transcription factor KUA1 OS=Arabidopsis thaliana ...	IPR001005: SANT/Myb
GK 14 days DS	Ca_05888	Ca6: 4088980 .. 4096217	KEA4_ARATH K(+) efflux antiporter 4 OS=Arabidopsis thaliana O…	IPR006153: Cation/H_exchanger
GK 14 days DS	Ca_05887	Ca6: 4099633 .. 4103060	TET2_ARATH Tetraspanin-2 OS=Arabidopsis thaliana OX=3702 GN=T..	IPR000301: Tetraspanin
GK 14 days DS	Ca_05886	Ca6: 4107411 .. 4108910	G7LJJ3_MEDTR Ectonucleotide pyrophosphatase/phosphodiesterase f...	IPR002591: Phosphodiest/P_Trfase
GK 14 days DS	Ca_05885	Ca6: 4110108 .. 4110886	PLA2A_ARATH Phospholipase A2-alpha OS=Arabidopsis thaliana OX=…	IPR013090: PLipase_A2_AS

GK 14 days DS	Ca_05884	Ca6: 4112113 .. 4121268	GALAK_ARATH Galacturonokinase OS=Arabidopsis thaliana OX=3702 ...	IPR000705: Galactokinase
GK 14 days DS	Ca_05883	Ca6: 4139456 .. 4139881	SAU23_ARATH Auxin-responsive protein SAUR23 OS=Arabidopsis tha.	IPR003676: SAUR_fam
GO 42 days DS	Ca_03159	Ca7: 3123388 .. 3123939	BIG1C_ARATH, Protein BIG GRAIN 1-like C OS=Arabidopsis thaliana…	
GO 42 days DS	Ca_03158	Ca7: 3129838 .. 3130206	C1K5D2_VIGRA (Chloroplast photosystem II subunit X OS=Vigna radiata	IPR009518: PSII_PsbX
GO 42 days DS	Ca_03157	Ca7: 3135386 .. 3135730	EF4L4_ARATH Protein ELF4-LIKE 4 OS=Arabidopsis thaliana	IPR009741: EARLY_FLOWERING_4_dom
GO 42 days DS	Ca_03156	Ca7: 3143105 .. 3143773	DABB1_ARATH Stress-response A/B barrel domain-containing prote...	IPR011008 Dimeric_a/b-barrel:
GO 42 days DS	Ca_03155	Ca7: 3147924 .. 3148592	UP3_ARATH Stress-response A/B barrel domain-containing prote...	IPR011008 Dimeric_a/b-barrel:

GO 42 days DS	Ca_03154	Ca7: 3155697 .. 3156812	TT2_ARATH Transcription factor TT2 OS=Arabidopsis thaliana	IPR001005: SANT/Myb
GO 42 days DS	Ca_03153	Ca7: 3160837 .. 3163988	MD20A_ARATH Mediator of RNA polymerase II transcription subuni	IPR013921: Mediator_Med20
GO 42 days DS	Ca_03152	Ca7: 3173129 .. 3183976	I1MSB4_SOYBN Uncharacterized protein OS=Glycine max PE=4 SV=2	IPR001194: cDENN_dom
GO 42 days DS	Ca_03151	Ca7: 3186039 .. 3188912	OPR3_ARATH 12-oxophytodienoate reductase 3 OS=Arabidopsis tha.	IPR001155: OxRdtase_FMN_N
GO 42 days DS	Ca_03150	Ca7: 3190936 .. 3195194	OFT35_ARATH O-fucosyltransferase 35 OS=Arabidopsis thaliana OX	IPR019378: GDP-Fuc_O-FucTrfase
GO 42 days DS	Ca_03149	Ca7: 3199251 .. 3201845	I1MSA9_SOYBN Uncharacterized protein OS=Glycine max PE=4 SV=1	IPR006553: Leu-rich_rpt_Cys-con_subtyp
GO 42 days DS	Ca_03148	Ca7: 3203022 .. 3203666	I3T4T4_MEDTR Uncharacterized protein OS=Medicago truncatula	

GO 42 days DS	Ca_03147	Ca7: 3206141 .. 3207838	I3S7S5_LOTJA Uncharacterized protein OS=Lotus japonicus	
GO 42 days DS	Ca_03146	Ca7: 3210207 .. 3211133	G7L7S5_MEDTR Putative uncharacterized protein OS=Medicago trunc...	
GO 42 days DS	Ca_03145	Ca7: 3217641 .. 3220736	I3SDT4_MEDTR Uncharacterized protein OS=Medicago truncatula	
GO 42 days DS	Ca_03144	Ca7: 3226163 .. 3229200	ALEUL_ARATH Thiol protease aleurain-like OS=Arabidopsis thalia...	IPR000169: Pept_cys_AS
GO 42 days DS	Ca_03143	Ca7: 3236202 .. 3238286	RIPK_ARATH Serine/threonine-protein kinase RIPK OS=Arabidopsi...	IPR000719: Prot_kinase_dom
GO 42 days DS	Ca_03142	Ca7: 3265637 .. 3267915	SBT18_ARATH Subtilisin-like protease SBT1.8 OS=Arabidopsis tha..	IPR000209: Peptidase_S8/S53_dom
GO 42 days DS	Ca_03141	Ca7: 3269912 .. 3271629	GG3_ARATH Guanine nucleotide-binding protein subunit gamma 3...	IPR015898: G-protein_gamma-like_dom

GO 42 days DS	Ca_03140	Ca7: 3284375 .. 3284857	G7JS83_MEDTR Putative uncharacterized protein OS=Medicago trunc	IPR012876: DUF1677_pln
GO 42 days DS	Ca_03139	Ca7: 3293784 .. 3297878	TBL16_ARATH Protein trichome birefringence-like 16 OS=Arabidop.	
GO 42 days DS	Ca_03138	Ca7: 3300073 .. 3305695	I1MS95_SOYBN Uncharacterized protein OS=Glycine max PE=4 SV=1	IPR002035: VWF_A
GO 42 days DS	Ca_03137	Ca7: 3306576 .. 3307399	G7JT65_MEDTR Uncharacterized protein OS=Medicago truncatula	IPR009327: Cupin_DUF985
GO 42 days DS	Ca_03136	Ca7: 3309557 .. 3312675	I3T5B9_MEDTR Uncharacterized protein OS=Medicago truncatula	IPR012762: Ubiq_biosynth_COQ9
GO 42 days DS	Ca_03135	Ca7: 3314839 .. 3316751	PRK3_ARATH Pollen receptor-like kinase 3 OS=Arabidopsis thali	IPR000719: Prot_kinase_dom

Notes. The reference genome and database used was CDC Frontier v1.0 in PulseDB. QTL is identified QTL named according to mapping family initials and DS phenotype. Name is the annotated gene name. Location is the genome browser location with Ca# identifying the chromosome and the next two numbers indicating the basepair start and end positions of the annotated gene. UniProt homology shows the top UniProt *Arabidopsis*

thaliana or other species match based on SwissProt or TrEMBL searches. The species codes are: ARATH = *Arabidopsis thaliana*; LOTJA = *Lotus japonicus*; MEDTR = *Medicago truncatula*; POPTR = *Populus trichocarpa*; SOYBN = *Glycine max*; VIGRA = *Vigna radiata*. The top InterPro annotation is given where available.
