# Supplementary material for: Inheritance of Early and Late Ascochyta Blight Resistance in Wide Crosses of Chickpea
Source: Genes (Basel). 2023 Jan 26;14(2):316. doi: 10.3390/genes14020316 (PMC9957483; doi:10.3390/genes14020316)
Supplement: Supplementary file 1 [file genes-14-00316-s001.zip › Supporting TableS2.pdf]

Table S2. Comparison of AUDPC of different sample types within the experiments for the Gokce x Oyali and Gokce x Karab families.

Comparison	Z statistic	p value	adj. pvalue
	Gockce x Oyali		
SusCheck - ResCheck	4.095135	4.22E-05	*4.22E-04*
SusCheck - Family	2.4100537	1.60E-02	*2.28E-02*
ResCheck - Family	-3.310279	9.32E-04	*2.33E-03*
SusCheck - Gokce	0.7149134	4.75E-01	5.27E-01
ResCheck - Gokce	-3.3802216	7.24E-04	*2.41E-03*
Family - Gokce	-1.4114194	1.58E-01	1.98E-01
SusCheck - Oyali	3.5953897	3.24E-04	*1.62E-03*
ResCheck - Oyali	-0.4997453	6.17E-01	6.17E-01
Family - Oyali	2.6122045	9.00E-03	*1.50E-02*
Gokce - Oyali	2.8804763	3.97E-03	*7.94E-03*
	Gokce x Karab		
SusCheck - ResCheck	4.46960898	7.84E-06	*7.84E-05*
SusCheck - Family	2.87995752	3.98E-03	*7.95E-03*
ResCheck - Family	-3.35502036	7.94E-04	*2.65E-03*
SusCheck - Gokce	2.04205022	4.11E-02	5.14E-02

ResCheck - Gokce	-2.42755876	1.52E-02	*2.53E-02*
Family - Gokce	-0.03135533	9.75E-01	9.75E-01
SusCheck - Karab	4.20624176	2.60E-05	*1.30E-04*
ResCheck - Karab	-0.26336722	7.92E-01	8.80E-01
Family - Karab	2.98763054	2.81E-03	*7.03E-03*
Gokce - Karab	2.16419154	3.04E-02	*4.35E-02*

Notes. Comparisons are the Gokce, Oyali, and Karab parents, SusCheck = susceptible check lines, ResCheck = resistant check lines, Family = F2 hybrids for each family. Summary Z statistics and p values for Kruskal-Wallis-Dunn post hoc tests between pairs of sample types are shown. P value adjustment by Benjamini-Hochberg method was applied. P values in italics show significant differences after adjustment.
