# Supplementary figures and images for: Inheritance of Early and Late Ascochyta Blight Resistance in Wide Crosses of Chickpea
Source: Genes (Basel). 2023 Jan 26;14(2):316. doi: 10.3390/genes14020316 (PMC9957483; doi:10.3390/genes14020316)

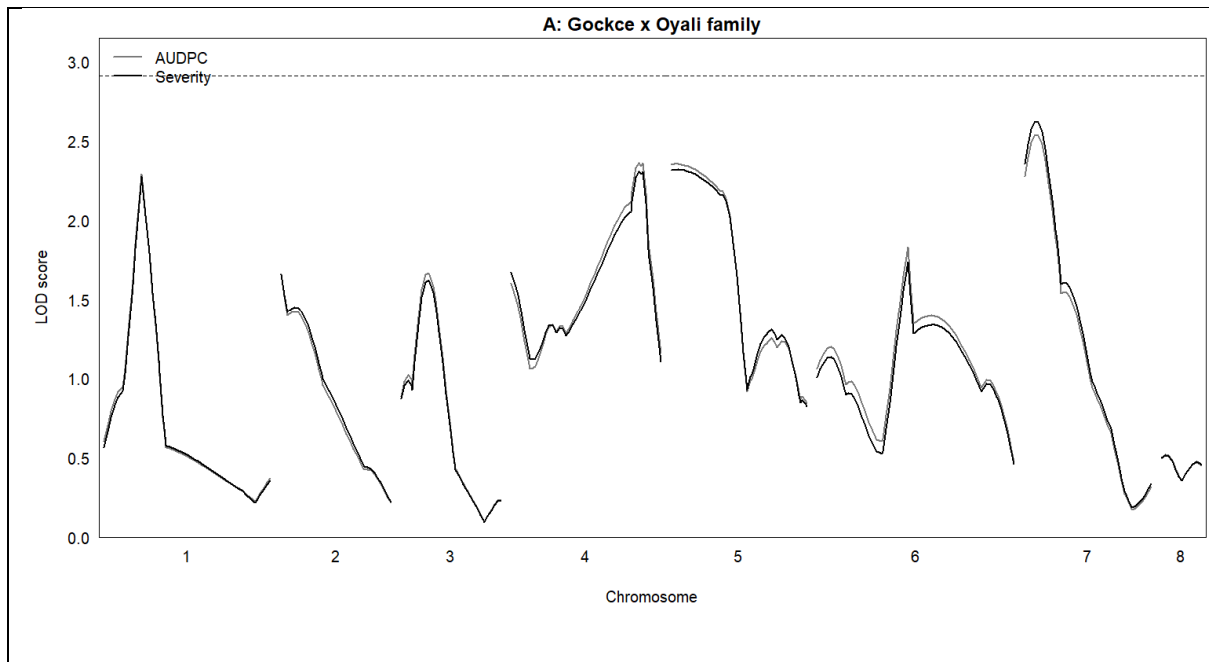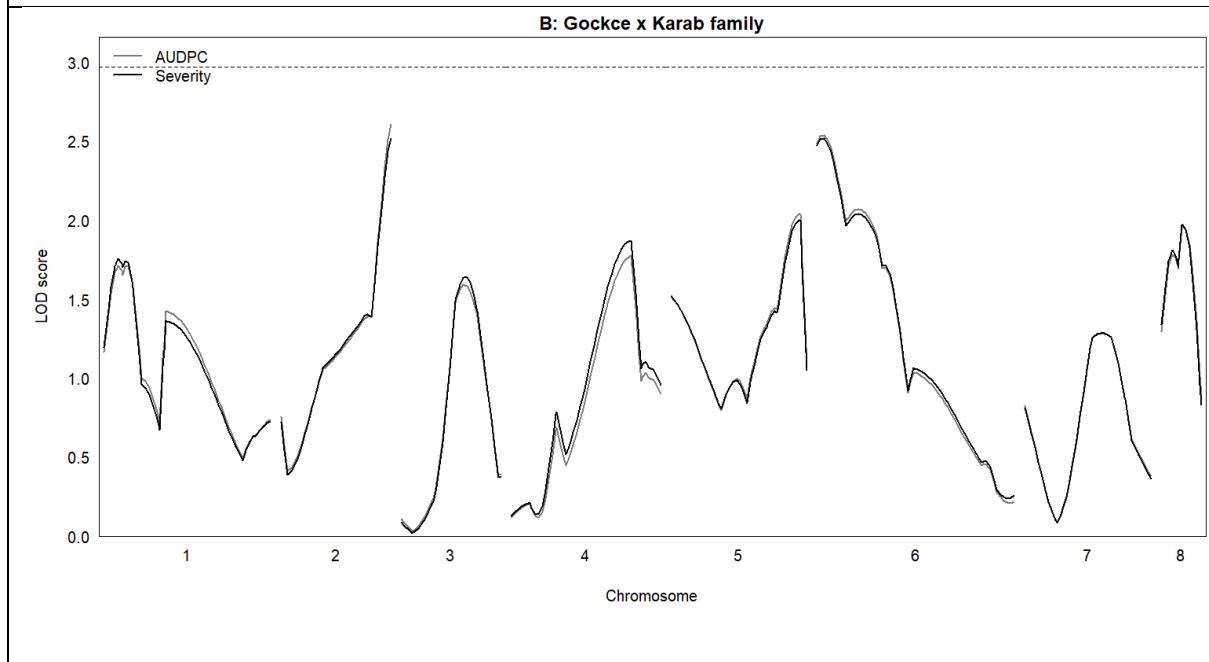

Supplement: Supplementary file 1 [file genes-14-00316-s001.zip › Supporting Figure S1.pdf]
